# Diagnostic significance of serum FGD5-AS1 and its predictive value for the development of cardiovascular diseases in patients with type 2 diabetes

**DOI:** 10.1186/s13098-022-00789-x

**Published:** 2022-01-28

**Authors:** Yongdi Wang, Jian Wang

**Affiliations:** 1 Department of Endocrinology, Weihai Municipal Hospital, Cheeloo College of Medicine, Shandong University, No. 70, Heping Road, Huancui District, Weihai, 264200 Shandong China; 2 Department of Laboratory, Yidu Central Hospital of Weifang, Weifang, Shandong China

**Keywords:** FGD5-AS1, Cardiovascular complications, Type 2 diabetes mellitus

## Abstract

**Background:**

As a result of the continuous rise in the incidence of type 2 diabetes mellitus (T2DM), related cardiovascular diseases (CVDs) have been a main healthy burden worldwide. This study aimed to investigate the potential role of FGD5-AS1 as a biomarker for the diagnosis of T2DM and predicting cardiovascular complications in T2DM.

**Methods:**

Three hundred subjects were recruited in this study, including 100 T2DM patients without CVDs, 100 T2DM patients with CVDs as well as 100 healthy subjects. Plasma FGD5-AS1 level was quantified using RT-qPCR assay. The correlation of FGD5-AS1 level with other key variables was assessed using Pearson correlation analysis. ROC curve analysis was performed to evaluate the diagnostic value of FGD5-AS1 for T2DM and related CVDs. The effect of FGD5-AS1 on AC16 and HA-VSMCs was determined.

**Results:**

FGD5-AS1 level showed a stepwise decrease in individuals with T2DM and CVDs compared to healthy persons. FGD5-AS1 was associated with BMI, systolic blood pressure, diastolic blood pressure, fasting glucose, 2-h postprandial blood glucose, HbA1c, triglycerides, usCRP, and HDL-cholesterol. The ROC analysis indicated FGD5-AS1 had a significant overall predictive ability to diagnose T2DM, T2DM with CVDs, and the combination of both. FGD5-AS1 increases the growth but alleviates apoptosis and fibrosis of high glucose-induced AC16 cells. FGD5-AS1 attenuate the growth and calcification but induced apoptosis of high glucose-treated HA-VSMC cells.

**Conclusions:**

These results suggest that FGD5-AS1 are associated with T2DM and measuring FGD5-AS1 could potentially contribute to T2DM screening and prediction for risk of cardiovascular complication.

## Background

Type 2 diabetes mellitus (T2DM) refers to a chronic health condition [1]. But it has reached alarming rates, with an incidence of 1 in 10, across the globe [2]. Now 537 million adults are living with diabetes, and this number would rise to 643 million by 2030 [3]. Cardiovascular diseases (CVDs) are common diabetes-related complications and contribute to more than one-half of all deaths in people affected by T2DM [4]. Atherosclerotic CVDs, such as coronary heart disease, cerebrovascular disease, and peripheral arterial disease, are the major cause of mortality from diabetes complications [5]. Heart failure is another main cause of mortality for individuals with diabetes. The 5-year mortality rate for diabetic patients after myocardial infarction, as high as 50%, is twice that of nondiabetic patients [6]. Controlling individual cardiovascular complications has shown great efficiency in preventing mortality or slowing disease progression in people with diabetes [7]. Risk factors for T2DM and the related microvascular complications comprise a combination of both genetic and metabolic factors [8]. Given the recognition of diabetes as the global burden and the risk of CVDs with diabetes, the urgent need for early detection of T2DM and effective prediction of CVDs in T2DM seems to be emerging.

Long noncoding RNAs (LncRNAs), a class of transcripts longer than 200 nucleotides, have no protein-coding potential [9]. However, they have been verified to implicate multiple cellular transcriptions, resulting in a profound impact on the dysregulation of growth-promoting, pro-fibrotic, and pro-apoptotic genes in target organs [10]. It is increasingly recognized that lncRNAs can actively participate in the pathogenesis of diverse metabolic and cardiovascular diseases, such as T2DM and CVDs [11, 12]. In recent years, lncRNAs have been widely investigated in various diseases, including T2DM, as a novel type of biomarkers. Li et al. has selected five upregulated lncRNAs as potential biomarkers by microarray analysis and verified the high diagnostic value of ENST00000550337.1 for pre-diabetes and T2DM [13]. Another example is HOTAIR, which is aberrant in serum of person with T2DM as a diagnostic factor for T2DM and a prediction for chronic complications of T2DM [14]. Considering that current treatment for many diabetic complications has not yet been fully effective, an exploration of epigenetic mechanisms may provide a novel and valuable insight into the pathophysiology of diabetic CVDs. For instance, recent advances in lncRNA DCRF have yielded information about its contribution to increasing cardiomyocyte autophagy in diabetic cardiomyopathy [15].

FGD5-AS1 has been associated with cardiovascular diseases [16, 17]. And a microarray profiling of muscle tissues from insulin-sensitive and insulin-resistant individuals showed that FGD5-AS1 was a susceptibility gene for T2D [18]. Herein, we determined the dysregulated expression of FGD5-AS1 in T2DM individuals, with or without CVDs, and investigated its diagnostic capability for T2DM and related CVDs.

## Methods

### Bioinformatic analysis

Dataset associated with lncRNA expression in T2D (GDS158) was obtained from Gene Expression Omnibus (GEO, https://www.ncbi.nlm.nih.gov/geo/) database.

### Study design and population

A total of 200 patients were recruited in this study, including 100 patients with T2DM without CVDs, and 100 patients with T2DM and progress in CVDs. In addition, 100 healthy subjects, who had no medical history of diabetes and CVDs, were recruited. The study protocol was approved by the scientific ethical committee of Weihai Municipal Hospital. Before inclusion into the study, written informed consent was obtained from each enrolled patient and healthy subject following the Declaration of Helsinki. T2DM was diagnosed according to criteria formulated by the American Diabetes Association. The diagnosis of CVDs was conducted following the current national guidelines by experienced cardiologists [19, 20].

### Measurement of plasma lncRNA/mRNA levels

Venous blood samples were drawn directly into Tempus™ Blood RNA Tube (Life Technologies, Austin, USA), and immediately vortex for 10 s. Then stabilized blood was transferred to 50-mL sterile conical tubes, and total RNA was isolated, purified, and stored by Tempus™ Spin RNA Isolation Kit (Life Technologies, Austin, USA) following the user guide. Extracted RNA was converted to cDNA by using the RT2 PreAMP cDNA Synthesis Kit (Qiagen). cDNA was synthesized from total RNA using HiScript II 1st Strand cDNA synthesis kit (Vazyme, China). The primers for qRT-PCR were: 5′-CGTGGAGAAGAATTGGGC-3′ (Forward) and 5′-CGTGGAGAAGAATTGGGC-3′ (Reverse) for FGD5-AS1; 5'-AACGGATTTGGTCGTATTG-3' (Forward) and 5'-GGAAGATGGTGATGGGATT-3' (Reverse) for *GAPDH*. qRT-PCR was performed with FastStart Universal SYBR Green Master (Rox) (Roche, Indianapolis, USA) at a 7500 Real-Time PCR System (Applied Biosystems, Waltham, USA). Data were analyzed by the 2^−ΔΔCt^ method normalized relative to the amount of *GAPDH*.

### Cell culture

AC16 (human primary ventricular cardiomyocytes) was purchased from Bluefcell (Shanghai, China) and cultured in DMEM (HyClone, Beijing, China) supplemented with 10% FBS (HyClone, Victoria, Australia). HA-VSMC (Human aortic vascular smooth muscle cell) was from Procell Life (Wuhan, China) and grown in its special medium (Procell Life). The culture condition for cells in the control group (mock) was routine, at 37 °C in 5% CO_2_. For the high glucose-induced group (HG), cells were culture in the presence of 30 mmol/L of glucose for an indicated time at 37 °C in 5% CO_2_.

Overexpressed FGD5-AS1 was produced with pcDNA3.1 termed as ov-FGD5-AS1 and the negative control pcDNA 3.1 vector (ov-NC) with inserted scrambled sequences were purchased from GenePharma (Shanghai, China.). The transfection was conducted by using Lipofectamine 3000 Reagent (Invitrogen, USA).

### Cell growth assay

For the viability experiment, Cell Counting Kit 8 (WST-8/CCK8) (ab228554, Abcam, Cambridge) was used. AC16 and HA-VSMC cells were seeded at a density of 4 × 10^3^ cells/well in 96-well plates. The number of the viable cells was determined at 0, 24, 48, 72 h according to the user guide.

### Calcification assays

HA-VSMC cells or transfected HA-VSMC cells were cultured with 30 mmol/L glucose for 14 days. Then, cells were washed, scraped into a solution, and lysed with Cell lysis buffer for Western and IP without inhibitors (Beyotime Biotechnology, Shanghai, China). Cell lysates were measured for alkaline phosphatase (ALP) activity using an Alkaline Phosphatase Assay Kit (Beyotime Biotechnology, Shanghai, China) and for osteocalcin secretion using an osteocalcin assay kit (Jiancheng, Nanjing). ALP activity and osteocalcin secretion were normalized to the total cellular protein of the cellular layers and then normalized to that of the control [21].

### Assessment of cell apoptosis and myocardial cell fibrosis

For apoptosis assay, non-transfected or transfected cells were cultured with 30 mmol/L glucose for 24 h. For assessment of cell fibrosis, AC16 cells were cultured with the complete medium including 30 mmol/L glucose for 48 h. Then, RNA in cells was extracted using Invitrogen TRIzol (California, USA). The expression of apoptosis markers, *caspase-3*, *Bcl-2,* and *Bax* mRNA, and fibrosis-related mRNAs (*Collagen-1* and *Collagen-3*) was checked by RT-qPCR using the 2^−ΔΔCt^ method.

### Statistical analysis

The normality of the data set was assessed by D’Agostino and Pearson omnibus normality test. Categorical variables were expressed as the number of cases and compared using the chi-square test. Continuous variables were shown as mean  ±  standard deviation (SD). The normally distributed continuous variables were analyzed using the student’s t test or one-way ANOVA as appropriate. Whereas non-normally distributed continuous variables were compared using the non-parametric Mann–Whitney U test or Kruskal–Wallis test as appropriate. *P* value of  < 0.05 was statistically significant. The correlation of FGD5-AS1 level with other key variables was accessed using Pearson correlation analysis. Receiver operating characteristic (ROC) curves were plotted to display the diagnostic value of the FGD5-AS1 level. Data statistical analyses were performed using IBM SPSS Statistics 23 or GraphPad Prism 7.

## Results

### Baseline characteristics of the participants

This study included three groups: 100 healthy subjects, 100 patients with T2DM but without CVDs, and 100 patients with T2DM and CVDs. The clinical and biochemical characteristics of all the participants are listed in Table 1. As shown, there is no difference in gender, age, and current smoking status among the three groups.

**Table 1:** Demographic and clinical characteristics of the study groups

Characteristics gender	Healthy subjects (*n* = 100)	T2DM without CVDs (*n* = 100)	*P* value (healthy subjects vs T2DM without CVDs)	T2DM with CVDs (*n* = 100)	*P* value (healthy subjects vs T2DM with CVDs)	*P* value (T2DM without CVDs vs T2DM with CVDs)
Male (*n*)	55	50	0.481	55	0.778	0.324
Age (years)	52.9 ± 6.9	54.3 ± 10.1	0.266	52.7 ± 10.0	0.865	0.200
BMI (kg/m^2^)	26.9 ± 2.7	28.5 ± 3.3	0.000	29.4 ± 3.3	0.000	0.036
Current smokers, *n*	29	32	0.651	35	0.366	0.651
Systolic blood pressure (mmHg)	121.3 ± 4.9	132.7 ± 8.3	0.000	139.8 ± 11.3	0.000	0.000
Diastolic blood pressure (mmHg)	75.2 ± 8.3	85.1 ± 9.0	0.000	93.2 ± 8.1	0.000	0.000
Fasting glucose (mmol/L)	4.6 ± 0.48	6.70 ± 0.9	0.000	8.1 ± 1.1	0.000	0.000
2-h postprandial blood glucose (mmol/L)	5.6 ± 0.9	8.5 ± 0.8	0.000	10.0 ± 1.7	0.000	0.000
HbA1c (%)	4.8 ± 0.5	6.1 ± 0.7	0.000	7.8 ± 0.9	0.000	0.000
Triglycerides (mmol/L)	1.2 ± 0.1	1.6 ± 0.2	0.000	2.1 ± 0.4	0.000	0.000
HDL-cholesterol (mmol/L)	1.3 ± 0.1	1.1 ± 0.2	0.000	1.0 ± 0.2	0.000	0.000
LDL-cholesterol (mmol/L)	3.2 ± 0.6	3.3 ± 0.6	0.348	4.1 ± 0.7	0.000	0.000
usCRP (mg/L)	2.8 ± 0.8	4.3 ± 1.8	0.000	6.1 ± 1.7	0.000	0.000

*T2DM* type 2 diabetes mellitus; *CVDs* cardiovascular disease; *BMI* body-mass index; *HDL-cholesterol* high-density lipoprotein cholesterol; *LDL-cholesterol* low-density lipoprotein cholesterol; *usCRP* ultra-sensitive C-reactive protein

### Level of FGD5-AS1 in blood and cells

**Fig. 1:**
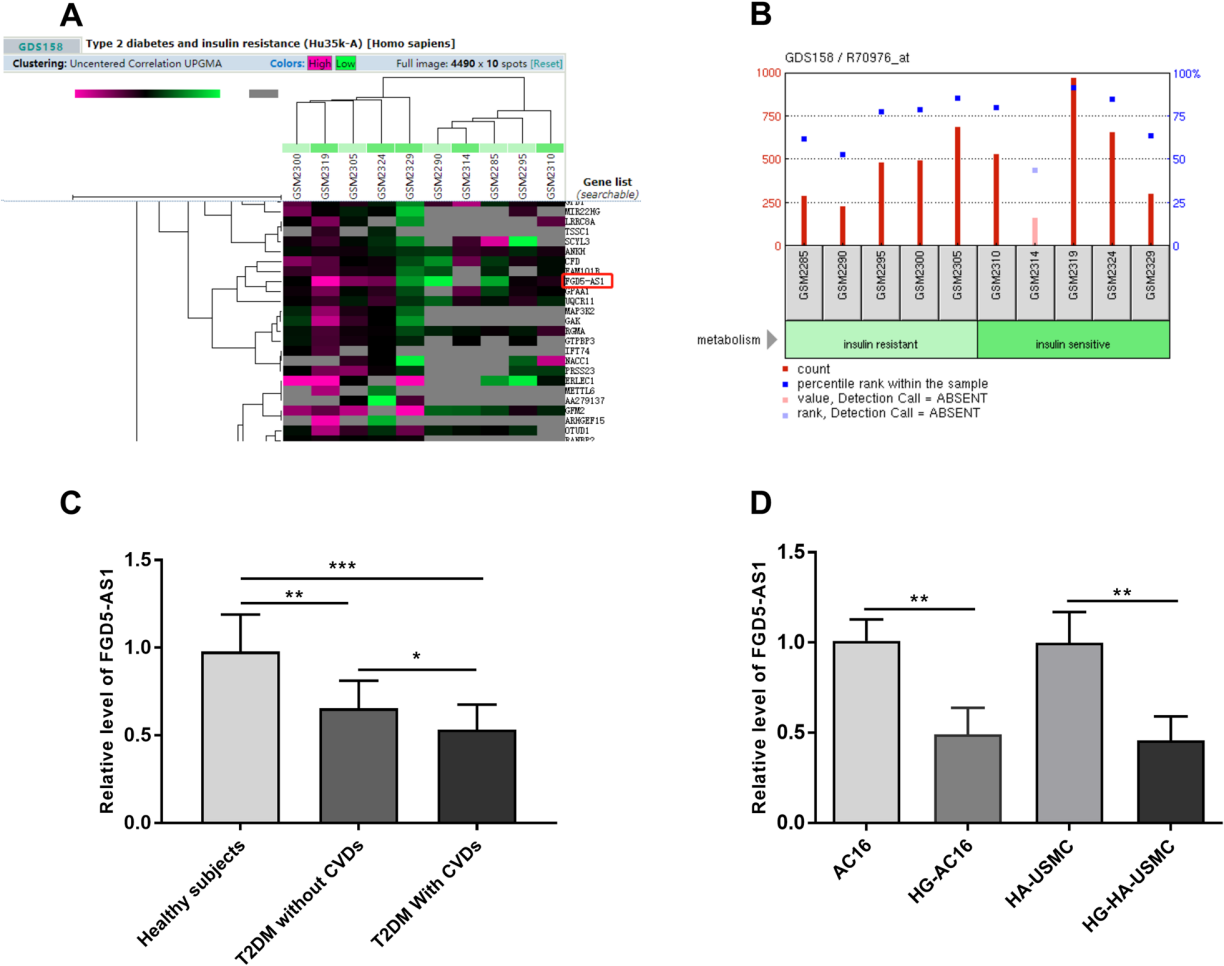
Relative expressions of FGD5-AS1. **A**, **B** Expression profiling of FGD5-AS1 from GEO DataSets (GDS158, a search for susceptibility genes for type 2 diabetes). **C** Relative expressions of FGD5-AS1 in healthy subjects, individuals with T2DM, and/no CVDs. **D** Relative expressions of FGD5-AS1 in normal and high glucose-induced AC16 and HA-VSMCs. *P  < 0.05, **P  < 0.01, *P  < 0.001. *T2DM* type 2 diabetes mellitus; *CVDs* cardiovascular disease; *HG* high glucose

The expression profiling of FGD5-AS1 from GDS158 DataSets showed that FGD5-AS1 was dysregulated in an insulin-resistant set (Fig. 1A, B). However, GEO DataSet (GDS158) was just a search for susceptibility genes for type 2 diabetes using insulin-sensitive and insulin-resistant instead of the CADs-related ones studied in this study. Then, the FGD5-AS1 level in the participants enrolled in this study was detected by RT-qPCR. As shown in Fig. 1C, the FGD5-AS1 level in the blood of T2DM patients was found to be significantly lower than that in healthy subjects (*P * < 0.01). FGD5-AS1 level in the blood from patients with T2DM and CVDs was lower than that in T2DM patients without CVDs (*P*  < 0.05) and that in healthy subjects (*P*  < 0.001). Furthermore, FGD5-AS1 levels were decreased in high-glucose-induced AC16 and HA-VSMC cells compared with those in normal-cultured cells (*P*  < 0.01; Fig. 1D).

### Correlation of FGD5-AS1 level with other variables

In T2DM individuals without/with CVDs, there were negative correlations between FGD5-AS1 level and BMI, systolic blood pressure, diastolic blood pressure, fasting glucose, 2-h postprandial blood glucose, HbA1c, and usCRP (*P*  < 0.01; Table 2). In contrast, FGD5-AS1 level was positively correlated with HDL-cholesterol levels in people with T2DM (*P*  < 0.01; Table 2).

**Table 2:** Correlation between plasma FGD5-AS1 level and other variables

Variables	Plasma FGD5-AS1 level			
	T2DM without CVDs		T2DM with CVDs	
	r	*P*	r	*P*
BMI	− 0.3601	0.0002	− 0.5283	< 0.0001
Systolic blood pressure	− 0.4862	< 0.0001	− 0.585	< 0.0001
Diastolic blood pressure	− 0.5012	< 0.0001	− 0.5463	< 0.0001
Fasting glucose	− 0.5803	< 0.0001	− 0.6405	< 0.0001
2-h postprandial blood glucose	− 0.57	< 0.0001	− 0.7257	< 0.0001
HbA1c	− 0.7389	< 0.0001	− 0.7988	< 0.0001
Triglycerides	− 0.1028	0.3086	− 0.1476	0.1429
HDL-cholesterol	0.2897	0.0035	0.4293	< 0.0001
LDL-cholesterol	− 0.4826	< 0.0001	− 0.1339	0.1840
usCRP	− 0.3595	0.0002	− 0.5882	< 0.0001

### Diagnostic value of FGD5-AS1 in individuals with or without T2DM

**Fig. 2:**
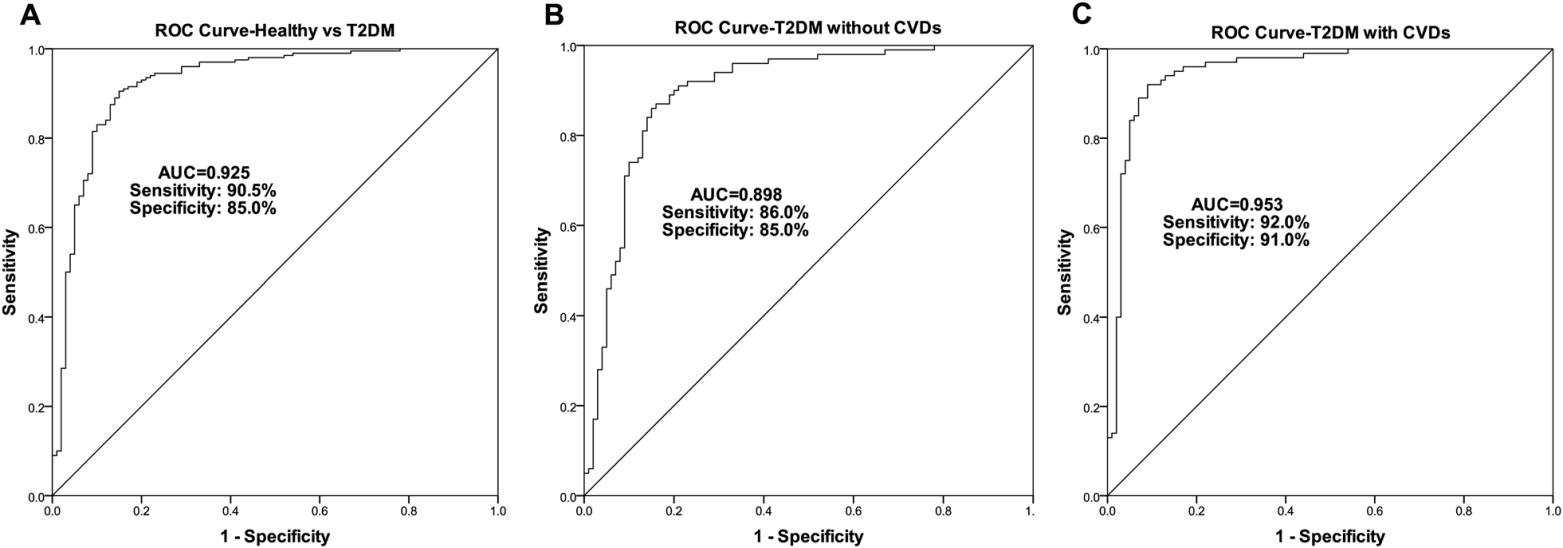
ROC curves were constructed for FGD5-AS1 to evaluate its diagnostic value for T2DM. **A** Healthy vs T2DM. **B** Healthy vs T2DM without CVDs. **C** Healthy vs T2DM with CVDs. *ROC* receiver operating characteristic; *T2DM* type 2 diabetes mellitus; *CVDs* cardiovascular disease

To evaluate the diagnostic value of FGD5-AS1 for T2DM, ROC curve analysis was performed. Figure 2 shows the sensitivity, specificity, and area under the curve (AUC). As shown in Fig. 2A, the ability of FGD5-AS1 was strong in discriminating individuals with or without T2DM (AUC 0.925; Sensitivity: 95.5%, Specificity: 85.0%). The ability of FGD5-AS1 was fair in discriminating healthy individuals and individuals with T2DM but without CVDs (AUC 0.898; Sensitivity: 86.0%, Specificity: 85.0%; Fig. 2B). The potential of FGD5-AS1 was excellent in discriminating healthy individuals and individuals with T2DM and CVDs (AUC 0.953; Sensitivity: 92.0%, Specificity: 91.0%; Fig. 2C).

### Diagnostic value of FGD5-AS1 in T2DM with or without CVDs

**Fig. 3:**
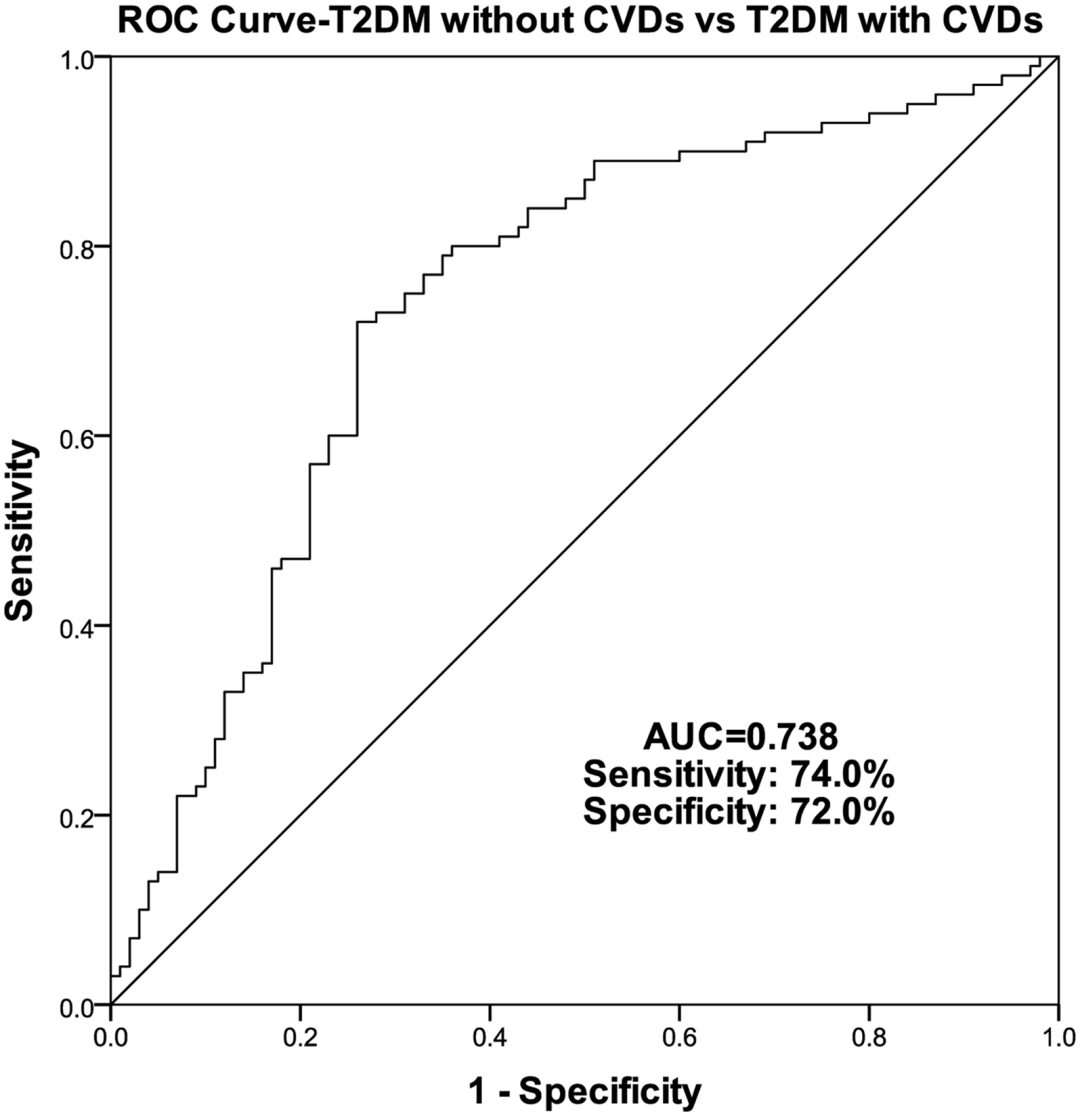
ROC was constructed for T2DM with CVDs as positive cases and T2DM without CVDs as negative cases

To have a better evaluation of the diagnostic value, the ROC curve was established to test the potential diagnostic value of FGD5-AS1 between T2DM with and without CVDs (Fig. 3). The AUC reached 0.738, with 74.0% sensitivity and 72.0% specificity, respectively.

### FGD5-AS1 alleviated apoptosis and fibrosis of high glucose-induced AC16 cells

**Fig. 4:**
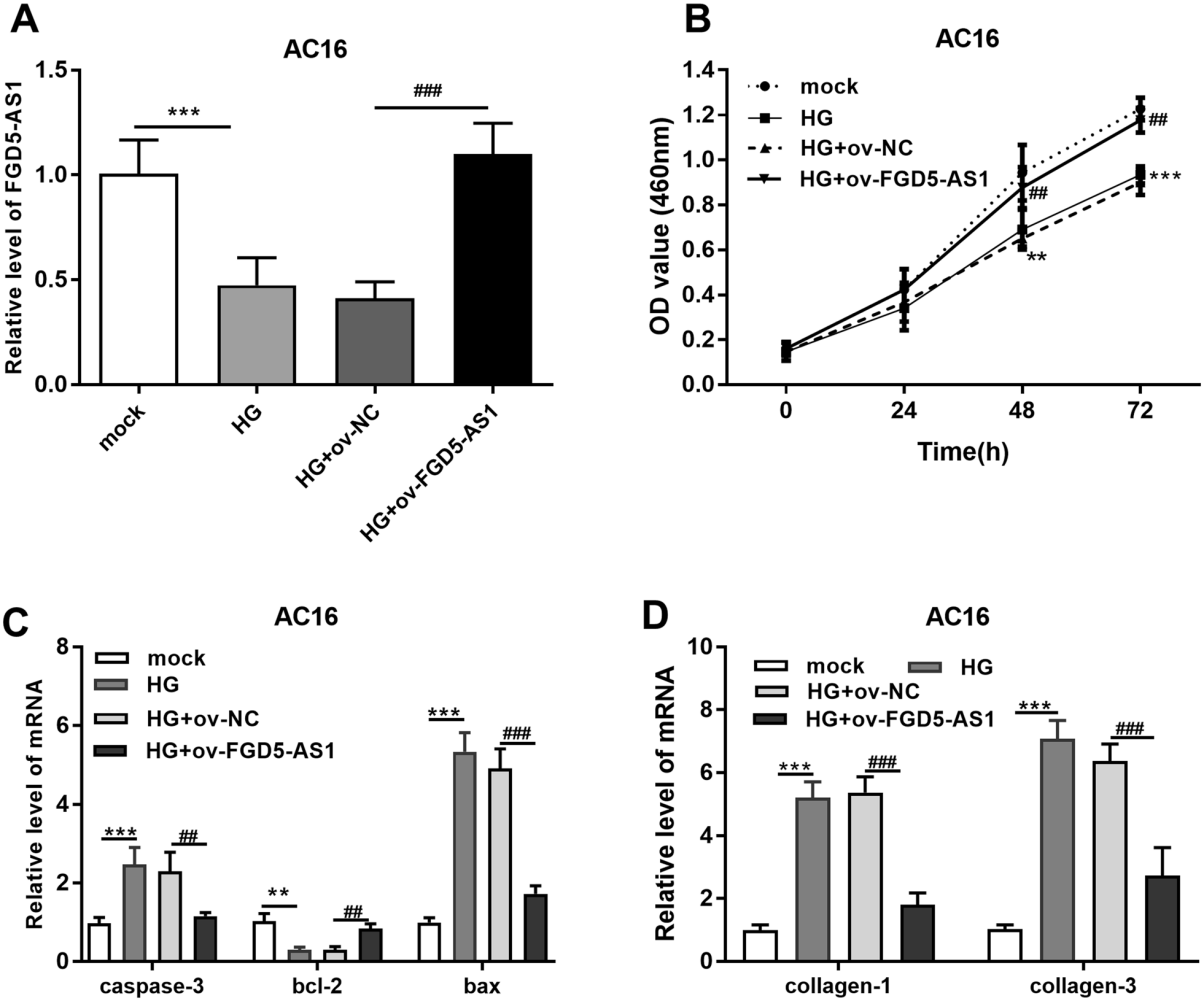
Overexpression of FGD5-AS1 boosted growth and alleviated apoptosis and myocardial fibrosis, of high glucose-induced AC16 cells. **A** The level of FGD5-AS1 was confirmed through RT-qPCR in different groups. **B** CCK-8 assay showed that upregulation of FGD5-AS1 reversed the reduction of cell proliferation in AC16 cells caused by high glucose. **C**, **D** The levels of caspase-3, bcl2, bax, collagen-1, and collagen-3 were detected through RT-qPCR in AC16 cells. **P  < 0.01, ***P  < 0.001 (compared with mock). ^##^*P*  < 0.01, ^###^*P*  < 0.001 (compared with HG  +  ov-NC). *HG* high glucose

To explore the role of FGD5-AS1 in AC16 cardiomyocytes, a gain-of-function approach was applied to overexpress FGD5-AS1 expression. RT-qPCR results showed that FGD5-AS1 was decreased after high-glucose induction but increased after transfecting with FGD5-AS1-overexpressed plasmid (*P * < 0.001; Fig. 4A). Cell growth assay showed high-glucose induction slow down the growth of AC16 cells, but FGD5-AS1 overexpression neutralize this effect (*P * < 0.01; Fig. 4B). The qRT-PCR results showed that the levels of mRNA of apoptosis-related genes (*caspase-3* and *bax*) and fibrosis-related genes (*collagen-1* and *collagen-3*) were prominently upregulated in high-glucose treated cells, but the FGD5-AS1 overexpression decreased them (*P*  < 0.01; Fig. 4C, D). These results indicated FGD5-AS1 has the potential to alleviate apoptosis and fibrosis in the high glucose-induced AC16 cells.

### FGD5-AS1 attenuated growth and calcification but induced apoptosis of high glucose-induced HA-VSMC cells

**Fig. 5:**
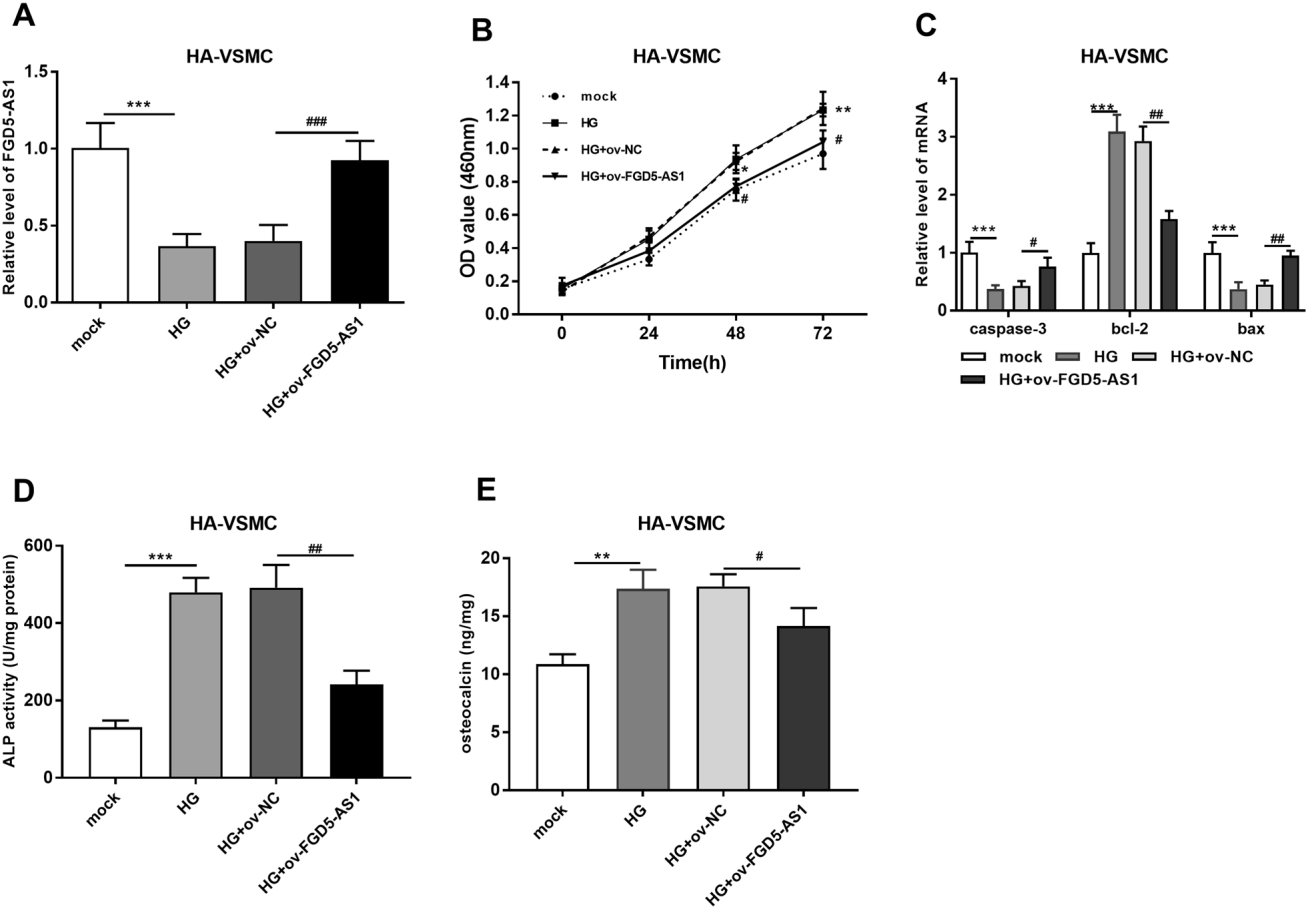
Overexpression of FGD5-AS1 boosted apoptosis and alleviated growth and calcification, of high glucose-induced HA-VSMC cells. **A** The level of FGD5-AS1 was confirmed through RT-qPCR in different groups. **B** CCK-8 assay showed that upregulation of FGD5-AS1 reversed the increase of cell proliferation in HA-VSMCs caused by high glucose. **C** Upregulation of FGD5-AS1 partly offset the change of apoptosis markers, caspase-3, Bcl-2 and Bax, caused by high glucose. **D**, **E** Upregulation of FGD5-AS1 reduced the increased level of calcification marker, alkaline phosphatase and osteocalcin. *P  < 0.05, **P  < 0.01, ***P  < 0.001 (compared with mock). ^#^*P*  < 0.05, ^##^*P*  < 0.01, ^###^*P*  < 0.001 (compared with HG  +  ov-NC). *HG* high glucose; *ALP* alkaline phosphatase

To clarify whether FGD5-AS1 is involved in apoptosis and calcification of HA-VSMC cells, an overexpression vector of FGD5-AS1 (ov-FGD5-AS1) or an empty negative control (ov-NCV) was transfected into HA-VSMC to regulate its expression. The results by RT-qPCR showed that FGD5-AS1 upregulation can mostly reverse the expression changes caused by high glucose (*P*  < 0.001; Fig. 5A). Furthermore, FGD5-AS1 upregulation could partly inhibit the increase of cell growth induced by high glucose (*P*  < 0.001; Fig. 5B). High glucose increased anti- apoptosis marker (bcl-2 mRNA) and reduced the apoptosis-related markers (*caspase-3* and *bax* mRNA), but FGD5-AS1 partly recovered them (*P * < 0.05; Fig. 5C). In addition, ALP activity and osteocalcin secretion were increased in high glucose-induced HA-VSMC but upregulation of FGD5-AS1 decreased these effects (*P*  < 0.05; Fig. 5D, E). Therefore, FGD5-AS1 could attenuate apoptosis and calcification of high glucose-treated HA-VSMC cells.

## Discussion

The ever-increasing incidence of diabetes worldwide has ratcheted up the risk for various associated complications, including CVDs. A high percentage of individuals with T2DM would develop CVDs, such as atherosclerosis, stroke and hypertension. Over the recent years, the trend in mortality and CVDs increased among both diabetics and non-diabetics. A greater emphasis on diabetic control and cardiovascular risk factors is most likely to reverse this encouraging trend. In the past decades, several common biochemical mechanisms and genetic factors have been implicated in the pathology of diabetes [22]. Recently, lncRNA-mediated mechanisms have also been implicated in T2DM and the progression of T2DM-related CVDs because crosstalk between the actions of various diabetogenic factors can amplify and perpetuate the expression of related genes [23]. Hence, further, unscramble of these novel epigenetic mechanisms could help improve the efficacy of early diagnosis and available therapies for T2DM and its subsequent CVDs.

In this study, FGD5-AS1 was identified as a lncRNA closely associated with T2DM and the development of CVDs. FGD5-AS1 has been previously detected in many cancers, such as glioblastoma, oral cancer [24–26]. It was also identified as one of eight candidate lncRNAs associated with dilated cardiomyopathy disease which is a common cause of heart failure [17]. In another lncRNA co-expression network analysis, FGD5-AS1 was differently expressed, associated with acute myocardial infarction-related signaling pathways, such as protein amino acid phosphorylation, regulating transcription, and signal transduction, and identified as a key lncRNA for acute myocardial infarction [27]. Here, we found FGD5-AS1 was downregulated in T2DM and T2DM-complicated CVDs, which was in line with the results from GEO DataSets. This dysregulation of FGD5-AS1 implies a potential role in T2DM and its related CVDs. Therefore, we tried to explore the diagnostic value of FGD5-AS1 in T2DM and its related CVDs. The correlation analysis revealed that FGD5-AS1 downregulation was closely interconnected with CVDs related factors, such as BMI, systolic blood pressure, diastolic blood pressure, fasting glucose, 2-h postprandial blood glucose, HbA1c, HDL-cholesterol, and usCRP.

ROC curves between healthy and T2DM with or without CVDs confirmed the great value of FGD5-AS1 in T2DM diagnosis, while the ROC curve between T2DM with CVDs and without CVDs showed distinguishing potential of FGD5-AS1 in CVDs progression. So FGD5-AS1 has potential in the diagnosis of T2DM and prediction of the CVDs.

FGD5-AS1 is an antisense RNA of FGD5, which belongs to the Rho guanine nucleotide exchange factor (Rho GEF) family complicating in various cellular processes [28]. FGD5-AS1 has been discovered to attenuate hypoxia-induced oxidative stress and apoptosis in human cardiomyocytes [29]. FGD5-AS1 has also exhibited an effect of extenuating oxygen–glucose deprivation and simulated reperfusion damage by increasing neuron proliferation but reducing neuron apoptosis [30]. In this study, FGD5-AS1 displayed an effect on the proliferation and apoptosis of human primary ventricular cardiomyocytes (AC16) and aortic vascular smooth muscle cells (HA-VSMC). Specifically, overexpressed FGD5-AS1 could accelerate growth and block apoptosis in AC16 cells, which may help relieve cardiomyocyte hypertrophy and apoptosis. FGD5-AS1 was also reported to increase the viability of C20/A4 cells but prevent apoptosis, thereupon then retard osteoarthritis development [31]. Combined with the report that FGD5‑AS1 modulated hypoxic injury in human cardiomyocytes partially via the miR‑195/RORA axis [29], it can be inferred that FGD5‑AS1 accelerate growth and block apoptosis of AC16 cells by moderating the miR‑195/RORA axis. For aortic vascular smooth muscle cells, FGD5-AS1 showed an inhibitory effect on cell growth and a promoting effect on apoptosis, which suggests that it plays an important role in cardiovascular diseases such as atherosclerosis. Moreover, overexpression of FGD5-AS1 can attenuate the calcification of HA-VSMCs induced by high glucose, as indicated by decreased ALP activity and OC secretion. Therefore, FGD5-AS1 exhibits a protective role in high glucose-induced injury. This supplied more support to the recognition of FGD5-AS1 as a predictive factor for T2DM-related CVDs.

In addition, FGD5-AS1 showed an additional effect on cell calcification and fibrosis. In high glucose-induced AC16 cells, FGD5-AS1 can reduce the fibrotic factors, indicating its anti-fibrosis role in cardiomyocytes. In high glucose-induced HA-VSMCs, FGD5-AS1 could reduce calcification stimulated by high glucose. The effect of FGD5-AS1 on vascular cells also existed in non-small cell lung cancer cells presenting as FGD5-AS1 downregulation inhibited HUVEC tube formation [32]. Our findings provide further insight into the function of FGD5-AS1 in VSMC calcification and cardiomyocytes’ fibrotic status under diabetic conditions.

## Conclusion

In summary, this work demonstrates that FGD5-AS1 is stepwise downregulated in T2DM and its related CVDs. Our findings indicate that routine measurement of FGD5-AS1 level in blood can improve the early diagnosis of T2DM and the prediction of CVDs in T2DM patients. The present study provides a preliminary elucidation of the clinical value of the FGD5-AS1 level in the risk assessment of CVDs in T2DM patients. These findings may provide a novel therapeutic approach for treating human CVDs related to diabetes.

## Data Availability

The datasets used and/or analysed during the current study are available from the corresponding author on reasonable request.

## Abbreviations

ALP: Alkaline phosphatase
AUC: Area under the curve
CCK8: Cell counting kit 8
CVDs: Cardiovascular diseases
LncRNAs: Long noncoding RNAs
NC: Negative control
ov-FGD5-AS1: Overexpression vector of FGD5-AS1
Rho GEF: Rho guanine nucleotide exchange factor
ROC: Receiver operating characteristic
SD: Standard deviation
T2DM: Type 2 diabetes mellitus
VSMC: Vascular smooth muscle cells
